# Identification and characterization of *ugpE* associated with the full virulence of *Streptococcus suis*

**DOI:** 10.1186/s13567-025-01513-z

**Published:** 2025-04-16

**Authors:** Qiulei Yang, Na Li, Yu Zheng, Yanyan Tian, Qiao Liang, Miaomiao Zhao, Hong Chu, Yan Gong, Tong Wu, Shaopeng Wei, He Wang, Guangmou Yan, Fengyang Li, Liancheng Lei

**Affiliations:** 1 State Key Laboratory for Diagnosis and Treatment of Severe Zoonotic Infectious Diseases, Key Laboratory for Zoonosis Research of the Ministry of Education, Institute of Zoonosis, and College of Veterinary Medicine, Jilin University, Changchun, China; 2 Department of First Hospital, Jilin University, Changchun, China; 3 College of Animal Science, Yangtze University, Jingzhou, China; 4 Department of Rehabilitation, The Second Hospital of Jilin University, Changchun, China

**Keywords:** *Streptococcus suis*, *ugpE*, virulence, ABC transporter, biofilm

## Abstract

*Streptococcus suis* (*S. suis*) is an emerging zoonotic pathogen that threatens both animal and human health worldwide. UgpE is a protein subunit of the Ugp (uptake of glycerol phosphate) transporter system that is involved in glycerophospholipid synthesis in bacterial membranes. In this study, an *ugpE* deletion mutant was constructed and the effects of *ugpE* deletion on cell morphology, biofilm formation, and virulence were investigated. Deletion of *ugpE* slowed down bacterial growth and impaired cell chain formation and capsular synthesis by downregulating the mRNA levels of the capsular regulon genes *cps-2B*, *cps-2C*, and *cps-2S*. Deletion of *ugpE* also led to decreased tolerance to heat, oxidative, and acid–base stress. Crystal violet staining and scanning electron microscopy demonstrate that *ugpE* may negatively regulate biofilm formation in liquid culture and the rdar biofilm morphotype on agar plates. Moreover, *ugpE* deletion not only reduced hemolysin activity, survival in whole human blood, and anti-phagocytosis ability against porcine alveolar macrophages (PAM) but also enhanced bacterial adhesion and invasion of human cerebral microvascular endothelial cells (hCMEC/D3) by upregulating the expression of multiple genes associated with cell adhesion. In a mouse infection model, *ugpE* deletion significantly attenuated virulence and lowered the number of viable bacteria in the blood and major organs, as well as distribution of macrophages. In conclusion, this study identified that UgpE may play a pivotal role in the regulation of various properties including virulence and biofilm formation of *S. suis*.

**Supplementary Information:**

The online version contains supplementary material available at 10.1186/s13567-025-01513-z.

## Introduction

*Streptococcus suis* (*S. suis*) is an important zoonotic pathogen that causes meningitis, septicemia, endocarditis, arthritis, and pneumonia in pigs, leading to huge economic losses to the pig industry. *S. suis* also infects humans through direct contact with pathogen-harboring droplets or wounds, causing diseases such as meningitis and toxic shock syndrome (STSS) that threaten human health. *S. suis* is classified into 29 serotypes (1–34, 1/2) according to its capsule antigen, among which *S. suis* serotype 2 (SS2) is the most prevalent and pathogenic strain in many countries, including China, Canada, and Brazil [1, 2]. In 1998 and 2005, outbreaks of human SS2 infection resulted in dozens of deaths in China [3]. In addition, SS2 has become the main pathogen causing meningitis in adults in southeast Asian countries and regions such as Vietnam, Thailand and Hong Kong [4–6].

The pathogenicity of *S. suis* depends on its virulence factor. To date, a variety of *S. suis* virulence factors have been identified, including capsular polysaccharide (CPS) [7], extracellular factor (EF) [8], muramidase-released protein (MRP) [9], suilysin (SLY) [10], glutamate dehydrogenase (GDH) [11], and fibronectin-binding protein (FbpS) [12, 13]. However, some experimental results indicate that the absence of one or more of these virulence or virulence-associated factors does not significantly affect the virulence of certain *S. suis* strains, suggesting that some of these virulence factors may act synergistically in pathogenicity or that new virulence or virulence-associated factors may be discovered. Therefore, there are still many unsolved aspects of the pathogenesis of *S. suis* that need to be further investigated to better prevent and control its associated diseases with *S. suis*.

ATP-binding cassette (ABC) transporters constitute a ubiquitous superfamily of integral membrane proteins that are responsible for the ATP-powered translocation of many substrates across membranes. Thus, they play vital roles in the regulation of a variety of physiological processes, including nutrient uptake, drug resistance, lipid transport, and the secretion of non-classical signaling molecules [14]. In prokaryotes, ABC transporters can take up nutrients, efflux toxins, and drugs, thereby affecting bacterial growth and enabling bacteria to resist various antibiotics. In addition, they play an important role in the interactions between pathogens and hosts. They are regarded as virulence factors in uropathogenic *Escherichia coli* because of their critical role in pathogenesis [15]. In *Streptococcus pneumoniae*, the ABC transporter BacA is required for the maintenance of chronic infections in mice [16]. In *S. suis*, proteome analysis has identified genes encoding diverse ABC transporters that may play roles in colonization and infection processes [17–20]. Moreover, the ABC transporter component ATPase MsmK regulates the pathogenicity of *S. suis* by utilizing carbohydrates [21]. Interestingly, evidence suggested that ABC transporters may also be involved in the regulation of biofilm formation in *Streptococcus mutans* [22].

The uptake of glycerol phosphate (Ugp) system belongs to the ABC transporter superfamily and is involved in glycerophospholipids (GPL) synthesis by uptake of *sn*-glycerol-3-phosphate (G3P) and certain glycerophosphodiesters (GPD) from the periplasm to the cytosol in *E. coli* [23]. This system is predicted to be prevalent in diverse bacteria but has been studied primarily in several Gram-negative bacteria including *E. coli* and *Thermus thermophilus* [24, 25]. In *E. coli*, it consists of four subunits known as the UgpABCE system, of which UgpA and UgpE constitute the transmembrane domains, UgpC forms a homodimer of the ATP-hydrolyzing subunit, and UgpB is a periplasmic substrate-binding protein [26]. Though also elucidated in *Mycobacterium tuberculosis* and *Corynebacterium glutamicum* [27, 28], its applicability to other Gram-positive bacteria remains uncertain.

GPL constitute the main components of bacterial cell membranes and play major roles in both cellular structure and function. In Gram-positive bacteria, GPL (phosphatidylglycerol and cardiolipin) increase the overall charge of the lipid membrane via aminoacylation which results in increased resistance to antibiotics, cationic antimicrobial peptides (CAP), and other cationic compounds [29]. Moreover, aminoacyl GPL also affect the rigidity, fluidity, and permeability of the membrane by neutralization into the membrane surface, and thereby impacting on basic cellular processes or tolerance to environmental challenges [30], such as swarming motility [31, 32], biofilm formation [33], and resistance to osmotic and acidic stresses [34–36]. Besides, GPL are required for the normal growth and virulence of *Mycoplasma pneumoniae* [37, 38]. In addition to GPL synthesis, Ugp transporters are also involved in the regulation of other cellular functions by the uptake of G3P and GPD. Excessive G3P accumulation causes growth defects in Gram-positive bacterium *C. glutamicum* and *M. tuberculosis* [39, 40]. Interestingly, Streptococci may incorporate phosphatidylcholine, a metabolite of glycerophosphocholine, into their membranes as a form of eukaryotic membrane mimicry to evade immune defenses such as CAP and low pH conditions after phagocytosis [41]. However, the role of Ugp transporters in the regulation of other cell functions, especially on virulence in other bacterial species remains unknown.

In this study, we assessed the role of *ugpE* (gene ID: YP_003028379.1), a homologous gene that encodes the Ugp transporter subunit UgpE (protein ID: ARL70172.1), in the regulation of various cellular phenotypes, including growth, stress tolerance, biofilm formation, and virulence of the zoonotic pathogen SS2. Our study contributes to elucidating the molecular mechanisms underlying the pathogenesis of *S. suis* and to the development of novel therapies and vaccines against diseases caused by *S. suis*.

## Materials and methods

### Bacterial strains and culture conditions

All strains used in this study are listed in Additional file 1. SS2 strain SC19 was kindly provided by Prof. Anding Zhang (Huazhong Agricultural University, Wuhan, China). SC19 was originally isolated from the brain of a sick pig during the epidemic outbreak in Sichuan province of China in 2005 and belongs to ST7 [42, 43]. The strain expresses muramidase-released protein, extracellular protein factor, and suilysin and is highly pathogenic to both mice and pigs [44]. Bacterial cultures were performed under different conditions according to the experimental requirements. SS2 and its derivatives were cultivated in Todd-Hewitt broth (THB; Hopebio, HB0311-3) or plated on THB agar plates supplemented with kanamycin (100 µg/mL; Beyotime, ST101) and spectinomycin (100 µg/mL; Macklin, S6106). *E. coli* strain DH5α was cultured in Luria–Bertani (LB; Becton Dickinson, DF0446-17-3) liquid medium or on LB agar plates containing 100 µg/mL spectinomycin if relevant.

### Construction of mutant and complemented strains

The *ugpE* chromosomal deletion mutant was constructed by homologous recombination as previously reported [45]. Briefly, the upstream and downstream homologous regions of *ugpE* were amplified using the A1/A2 and A3/A4 primer pairs, resulting in two fragments that were fused and ligated into the pSET4s shuttle vector [46]. The recombinant plasmid was electroporated into SC19 cells, and the bacteria were selected on THB agar plates supplemented with 100 μg/mL spectinomycin at 37 °C. Subsequently, the colonies grown on the plate were inoculated and cultured in THB without spectinomycin at 28 °C to screen double-crossover mutants. The mutant strains (Δ*ugpE*) were spectinomycin susceptible and confirmed by PCR using the primer pair A5/A6. Gene complementation was conducted by cloning *ugpE* into the pSET2 shuttle vector [46] using primer Y1/Y2, which contains a putative promoter region. The inserted DNA sequences were confirmed using PCR and DNA sequencing. The recombinant plasmid was electroporated into the mutant strain to produce the complemented strain CΔ*ugpE*. The plasmids and primers used are listed in Additional files 1 and 2, respectively.

### Growth curves

Briefly, *S. suis* (WT, Δ*ugpE*, and CΔ*ugpE*) cells were cultured overnight, diluted 1:100, and grown to the exponential phase in THB (with 10% FBS). The bacteria were diluted in THB again at 1:100, adjusted to OD_600_ = 0.01, and incubated at 37 °C under vigorous shaking at 180 rpm. The OD_600_ value was measured every hour for 15 h. Growth curves were plotted using GraphPad Prism (version 9.0; San Diego, CA, USA).

### Observation of colony morphology

The bacterial chain length and capsule were observed using light and transmission electron microscopy (TEM), respectively. Briefly, a suspension of bacterial cells (WT, Δ*ugpE*, CΔ*ugpE*) grown overnight was diluted in THB (with 10% FBS) medium and were diluted in THB again at 1:100 dilution and incubated at 37 °C under vigorous shaking at 180 rpm. For light microscopy, the cells were harvested at the mid-log growth phase for Gram staining. The bacterial chain length was observed under a light microscope (Nikon) and determined by counting the cell numbers of 200 chains per cell. For TEM, procedures were followed as previously described with slight modifications [47, 48]. Briefly, cells in the mid-log growth phase were collected and pre-fixed in 2.5% glutaraldehyde containing 100 mM lysine overnight at 4 °C. After washing twice with PBS, the samples were post-fixed with 1% osmium tetroxide in PBS for 1–2 h at room temperature. After dehydration with graded series of ethanol and propylene oxide, the samples were embedded in epoxy resin, sectioned and stained with 1% uranyl acetate and alkaline lead citrate. The samples were observed under a Hitachi HT 7700 (Hitachi, Tokyo, Japan) electron microscope at 80 kV. The bacterial cell width, cell wall, and capsule thickness were determined using ImageJ software (version 1.8.0) by randomly selecting 20 cells.

### Bacterial surface hydrophobicity test

Bacterial surface hydrophobicity test was performed by measuring adsorption to* n*-hexadecane as previously described [49].

### Bacterial stress tests

Bacterial stress tests were performed as previously described with slight modifications [50]. Briefly, overnight cultures of *S. suis* cells (WT, Δ*ugpE*, CΔ*ugpE*) were diluted in THB medium at 1:100 dilution and grown to mid-log stage at 37 °C. Cell samples from each group were collected and OD_600_ values were measured and adjusted to OD_600_ = 0.1 and grown to the mid-log stage. For the hydrogen peroxide (H_2_O_2_) test, H_2_O_2_ (30 mM) and PBS (control) were added to the cells and incubated at 37 °C for 20 min. For the heat stress analysis, cells were incubated at 37 °C and 40 °C for 12 h. For the acid tolerance test, cells were harvested after centrifugation at 4 °C, washed three times with PBS (pH 7.4), and cultured with fresh THB medium at different pH values (2.0, 4.0, 6.0, 7.0, 8.0, 10.0, and 12.0) for 45 min at 37 °C. For all assays, cells were plated on THA plates after serial dilution in PBS and bacterial numbers were counted after overnight incubation. Each sample was set up in triplicate and repeated independently three times.

### Hemolytic assay

The hemolytic activity was detected as previously described [51]. Briefly, overnight cultures of *S. suis* cells (WT, Δ*ugpE*, CΔ*ugpE*) were diluted in THB medium at 1:100 dilution and grown to mid-log stage at 37 °C. Cells were removed by centrifugation and 100 µL of the supernatant were mixed with equal volume of 2% sheep erythrocyte suspension (Yuanye Bio-Technology, R22332) in a 96-well microplate. After incubation for 2 h at 37 °C, the mixture was centrifuged at 1000 × *g* for 10 min to remove unlysed erythrocytes and the supernatants (100 μL) were transferred to a new 96-well microplate to measure the OD_540_ value. Each sample was set up in triplicate and repeated independently three times.

### Bacterial adhesion and invasion assays

Adhesion and invasion assays were performed in the human cerebral microvascular endothelial cell line, hCMEC/D3, as described previously [52]. hCMEC/D3 cell line was kindly provided by Prof. Yan Chen (College of Life Sciences, Jilin University, Changchun, China). Briefly, hCMEC/D3 cells were cultured to approximately 2.5 × 10^5^ cells/well in a 24-well plate, followed by infection with different groups of bacteria at a multiplicity of infection (MOI) of 100:1. After incubation at 37 °C and 5% CO_2_ for 1 h, the cells were washed four times with PBS to remove non-adherent bacteria. For the adhesion assay, cells were lysed with 0.1% saponin. The levels of adhesion were expressed as the total number of CFU recovered per well. For the invasion assay, cells were incubated with fresh DMEM supplemented with 5 μg/mL penicillin G (Sangon Biotech; B540729-0010) and 100 μg/mL gentamicin (Sangon Biotech; E607063-0100) for 2 h to kill extracellular and surface-adherent bacteria. The cells were then washed three times with PBS and lysed with 0.1% saponin (Merck; 47036). Lysates were plated on THA plates after serial dilution in PBS and bacterial numbers were counted after overnight incubation. To confirm that all of the extracellular bacteria were killed after the antibiotic treatment, a 100 μL sample of the last PBS wash solution was plated on THB agar (data not shown). The levels of invasion were expressed as the total number of CFU recovered per well. Each sample was set up in triplicate and repeated independently three times.

### Phagocytosis

The phagocytosis assay was conducted in porcine alveolar macrophages (PAM) as described previously, with slight modifications [53]. Immortalized PAM (ATCC, CRL-2845) was provided by the Harbin Veterinary Research Institute of the Chinese Academy of Agricultural Sciences (Harbin, China). PAM cells were cultured to approximately 2.5 × 10^5^ cells/well in a 24-well plate, followed by infection with different groups of bacteria at a MOI of 40:1. After incubation at 37 °C and 5% CO_2_ for 1 h, the cells were washed three times with PBS and incubated with fresh DMEM supplemented with 5 μg/mL penicillin G and 100 μg/mL gentamicin for 2 h to kill extracellular and surface-adherent bacteria. The cells were then washed three times with PBS and lysed with trypsin (ThermoFisher; R001100). Lysed cells were serially diluted and plated on THA plates for counting. Each sample was set up in triplicate and repeated independently three times.

### Whole blood survival

Each group of bacteria (WT, Δ*ugpE*, CΔ*ugpE*) was cultured in THB supplemented with 1% glucose until mid-log growth phase. After centrifugation, the bacterial body was collected and resuspended to an OD_600_ of 0.2 in PBS. Then, 1 mL of germ-free human whole blood containing EDTA-Na_2_ anticoagulant (Iphase, 033A13.120) was added to 100 μL of the bacterial samples and incubated at 37 °C for 2 h. The mixture was serially diluted and plated on THA plates for cell counting. Survival was calculated as follows: (recovered CFU/CFU in the original inoculum) × 100%. The experiment was repeated three times, and three samples were collected in triplicate each time.

### Biofilm formation

Biofilm formation (adherence to the cell wall) was assessed as described previously, with slight modifications [54]. Briefly, each group of bacteria (WT, Δ*ugpE*, CΔ*ugpE*) was incubated in 96-well plates with 200 μL THB medium per well at 37 °C for 24 h, 48 h, and 72 h. After removing the unattached bacteria, adherent cells were stained with 0.2% crystal violet, washed, and dissolved in a 95% alcohol solution. Biofilms were measured as the absorbance of dissolved crystal violet at OD_595_. Rdar (red, dry, and rough) morphotype assessment was performed as described previously [55]. Briefly, 5 µL of an overnight culture of OD_600_ = 5 suspended in water was spotted onto THA plates containing the dye Congo red (40 µg/mL) and Coomassie Brilliant Blue G-250 (20 µg/mL) or calcofluor white (Fluorescence Brightener 28, 50 µg/mL). After incubation at 37 °C for 24 h, 48 h, and 72 h, pictures were taken to analyze the development of the colony morphology structure and dye binding. The surface morphology of the colonies was plotted and analyzed using the ImageJ software (version 1.8.0).

### Scanning electron microscopy

Scanning electron microscopy (SEM) was performed to visualize the macrocolony biofilms. An overnight culture of OD_600_ = 5 suspended in water was prepared and spotted on THA plates pre-embedded with a rectangular glass slide. After 48 h of culture at 37 °C, the macrocolonies on the glass slide were fixed in fixative solution (0.5% glutaraldehyde and 2.5% paraformaldehyde in 10 mM HEPES, pH 7.0), dehydrated with acetone, sputter-coated with gold/palladium, and observed using a Zeiss Merlin field emission SEM at an acceleration voltage of 10 kV with an Everhart–Thornley secondary emission (SE) detector.

### RNA isolation and qRT-PCR

Each group of bacterial cells (WT, Δ*ugpE*, CΔ*ugpE*) was grown in THB medium until the mid-log growth phase. Total RNA was extracted using TRIzol reagent (Invitrogen, USA), according to the manufacturer’s instructions. RNA was treated with DNase (Invitrogen, USA), and RNA quality was assessed by gel electrophoresis and PCR. The RNA concentration was measured using a NanoDrop 2000 system (Thermo Scientific). cDNA was synthesized using 1 μg of RNA from each sample using the Prime Scrip RT Master Mix Kit (TaKaRa, Dalian, China). Quantitative PCR (qPCR) was performed using TB Green PCR Master Mix (TaKaRa) on a Quantagene q225 qPCR System (Kubo, Beijing, China). The 16S rRNA gene was used as an internal reference gene for normalization. Data were analyzed using the 2^−ΔΔCT^ method. All experiments were performed at least three times. The primers used are listed in Additional file 2.

### Animal experiments

All animal experiments were approved by the Institutional Animal Care and Use Committee of Jilin University and were conducted in accordance with the Chinese Laboratory Animal Administration Act 1988. To determine bacterial virulence in mice, forty 6-week old female BALB/c mice (body weight 18 ± 2 g, purchased from Changsheng Biotechnology Co., Ltd., China) were divided into four groups (10 mice per group) randomly and intraperitoneally infected with 100 μL (1 × 10^9^ CFU, LD_50_ = 5 × 10^8^ CFU) of SC19, Δ*ugpE*, CΔ*ugpE* or PBS (as control). All mice were housed in standard plastic mouse cages and kept under constant room temperature (23 ± 3 °C), humidity (55 ± 5%), and observed for one week. Clinical scores, body weight changes, and animal deaths were recorded every 12 h.

For histopathological analysis, mice were intraperitoneally infected with 100 μL (5 × 10^8^ CFU) of each strain or PBS (as a control). After 3 days, the mice were euthanized by inhalation of CO_2_ and organs including the brain, lung, liver, spleen, and kidney were extracted and frozen at − 80 °C or used immediately for histopathological analysis and flow cytometry. Histopathological analysis was performed as described previously [56]. Briefly, the tissues were fixed in 10% formaldehyde solution, embedded in paraffin, sliced, and stained with hematoxylin and eosin (HE). The stained sections were visualized, and pathological changes in the tissues were analyzed under an optical microscope (Olympus, Tokyo, Japan). Additionally, to evaluate the distribution of bacteria in different organs after infection, mice were intraperitoneally infected with 100 μL (2 × 10^8^ CFU) of SC19 and Δ*ugpE*, respectively. After 3 days post-infection, the mice were euthanized and equal weights of brain, lung, liver, spleen, kidney, and blood samples were collected and homogenized. The numbers of viable bacteria recovered from the organs and blood were counted after overnight incubation at 37 °C.

### Flow cytometry

Macrophages in the lung, liver, spleen, heart, and kidney were assessed using flow cytometry, as previously reported [57]. Briefly, the organs were shred and digested in HBSS containing gelatinase A (100 U/mL) and DNase (20 µg/mL). After filtration and rinsing with PBS, the samples were centrifuged and the pellets were treated with ice-cold RBC lysis buffer for 5 min. The isolated cells were washed with ice-cold PBS again and centrifuged to collect the cell pellets for antibody labeling in a fluorescent washing buffer (Thermo Fisher). The cell suspension was incubated with BV421 anti-mouse CD11b (Biolegend, 101236), APC anti-mouse F4_80 (Biolegend, 122115), PE anti-mouse CD206 (Biolegend, 141705), PerCP/Cy5.5 anti-mouse CD45 (Biolegend, 103132), and PE-Cy7 CD86 anti-mouse antibodies (Biolegend, 105013) in the dark for 30 min. After centrifugation, washing with PBS, and resuspension in FWB, the same number of cells in each group was processed with a flow cytometer (CytoFLEX) and analyzed with FlowJo software (Version 10.4).

### Statistical analysis

The experimental data were statistically analyzed using GraphPad Prism (version 9.0, San Diego, CA, USA). Data are presented as mean ± SD from three independent replicates. Differences between the mean values of normally distributed data were assessed using one-way ANOVA (Dunnett test). *P*-value < 0.05, as indicated by “*”. *P*-value < 0.01 indicates that the difference is extremely significant, as indicated by “**”.

## Results

### *ugpE* deletion inhibited chain formation and capsular synthesis of SS2

**Figure 1:**
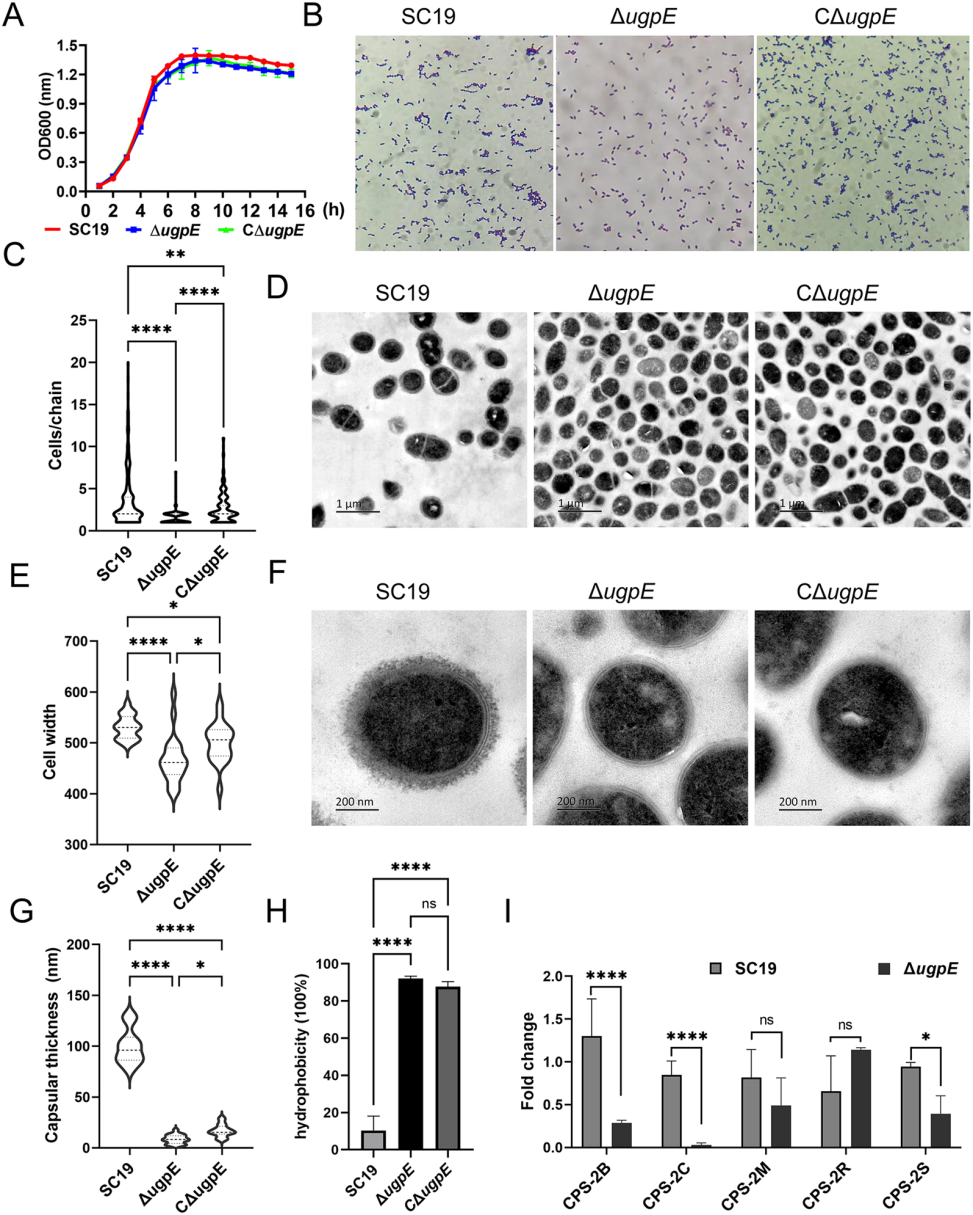
***ugpE*** **deletion inhibited chain formation and capsular synthesis of SS2.** **A** Growth kinetics of SC19 wild-type, Δ*ugpE* and CΔ*ugpE* at 37 °C. **B** Observation of cell morphology by Gram staining and light microscopy (1000 ×). **C** Analysis of cell number per chain in each group of cells. The quantification is based on results from at least three independent experiments with the assessment of 200 cells from each group. **D** Observation of cell morphology by transmission electron microscopy (TEM). Size bar, 1 μm. **E** Analysis of cell width in each group of cells. The quantification is based on results from at least three independent experiments with the assessment of 20 cells from each group. **F** Observation of capsule structure by TEM. Size bar, 200 nm. **G** Analysis of capsular thickness in each group of cells. The quantification is based on results from at least three independent experiments with the assessment of 20 cells from each group. **H** Determination of hydrophobicity of wild-type and mutant strains using *n*-hexadecane. **I** Analysis of mRNA levels of *cps* regulon genes *cps-2B*, *cps-2C*, *cps-2S*, and *cps-2 M* by qRT-PCR. The data are presented as mean ± SD. Data were analyzed using one-way ANOVA (Dunnett test) (**C, E, G, H**) and paired *t* tests (**I**), respectively. ***P < 0.001; **P < 0.01; *P < 0.05. ns, not significant.

We first constructed an isogenic *ugpE* deletion mutant strain (Δ*ugpE*) by homologous recombination and a corresponding complemented strain (CΔ*ugpE*) (Additional file 3). The effect of *ugpE* on the growth kinetics were examined. Overall, the Δ*ugpE* mutant appeared to grow more slowly than the SC19 wild-type, as observed by the growth curve, but the differences between each group were not statistically significant in the first 10 h; however, a significant growth defect was observed after 10 h of culture during the late stational and death phase (Figure 1A; Additional file 4A). Interestingly, observation of cell morphology by Gram staining and light microscopy revealed that the Δ*ugpE* mutant displayed abnormal cell chain formation compared to the wild-type (Figures 1B and C). After 72 h of culture in THB medium, the majority of the bacterial chains of the SC19 wild-type consisted of 1–4 cell (s) per chain (approximately 80%), while the rest consisted of more than 5 cells per chain, and some even displayed very long chains with more than 10 cells, which was not observed in the Δ*ugpE* mutant (Figure 1C; Additional files 4B and C). On the contrary, most of the Δ*ugpE* mutants consisted of only 1–3 cell (s) (approximately 95%), and bacteria consisting of more than 4 cells only accounted for a minor proportion. The majority of the complemented strain CΔ*ugpE* also consisted of 1–4 cell (s) (approximately 70%), but no cells with long chains (> 10 cells per chain) were found (Figure 1C; Additional file 4C). Moreover, deletion of *ugpE* also led to a significantly decreased cell width compared to the SC19 wild-type, as confirmed by transmission electron microscopy (TEM) (Figures 1D and E). Overall, these results suggest a potential role for *ugpE* in regulating SS2 chain formation.

Of note, TEM also shows that the SC19 wild-type had a thick capsule structure, while the capsule structure of Δ*ugpE* was hardly observed (Figure 1F; Additional file 4D). In line with this, the capsular thickness of the Δ*ugpE* strain was significantly thinner than that of the SC19 wild-type and complemented strain CΔ*ugpE* (Figure 1G). In contrast, cell wall thickness shows no obvious differences among the groups (Additional file 4E). Moreover, the Δ*ugpE* strain displays a significantly increased surface hydrophobicity (an indicator of encapsulation [58]) compared to the SC19 wild-type (Figure 1H). Knowing that the bacterial capsule structure was abolished, we assessed the level at which *ugpE* affected the capsular regulon genes. The *cps* gene cluster of SS2 comprises 25 genes [59] and we chose several of them for qRT-PCR analysis. Compared to the SC19 wild-type, the mRNA levels of *cps-2B*, *cps-2C*, and *cps-2S* were downregulated, while the mRNA levels of *cps-2 M* and *cps-2R* were not affected (Figure 1I). Overall, these results indicate that *ugpE* seems to affect capsule synthesis of SS2.

### *ugpE* deletion led to decreased tolerance to environmental stresses

**Figure 2:**
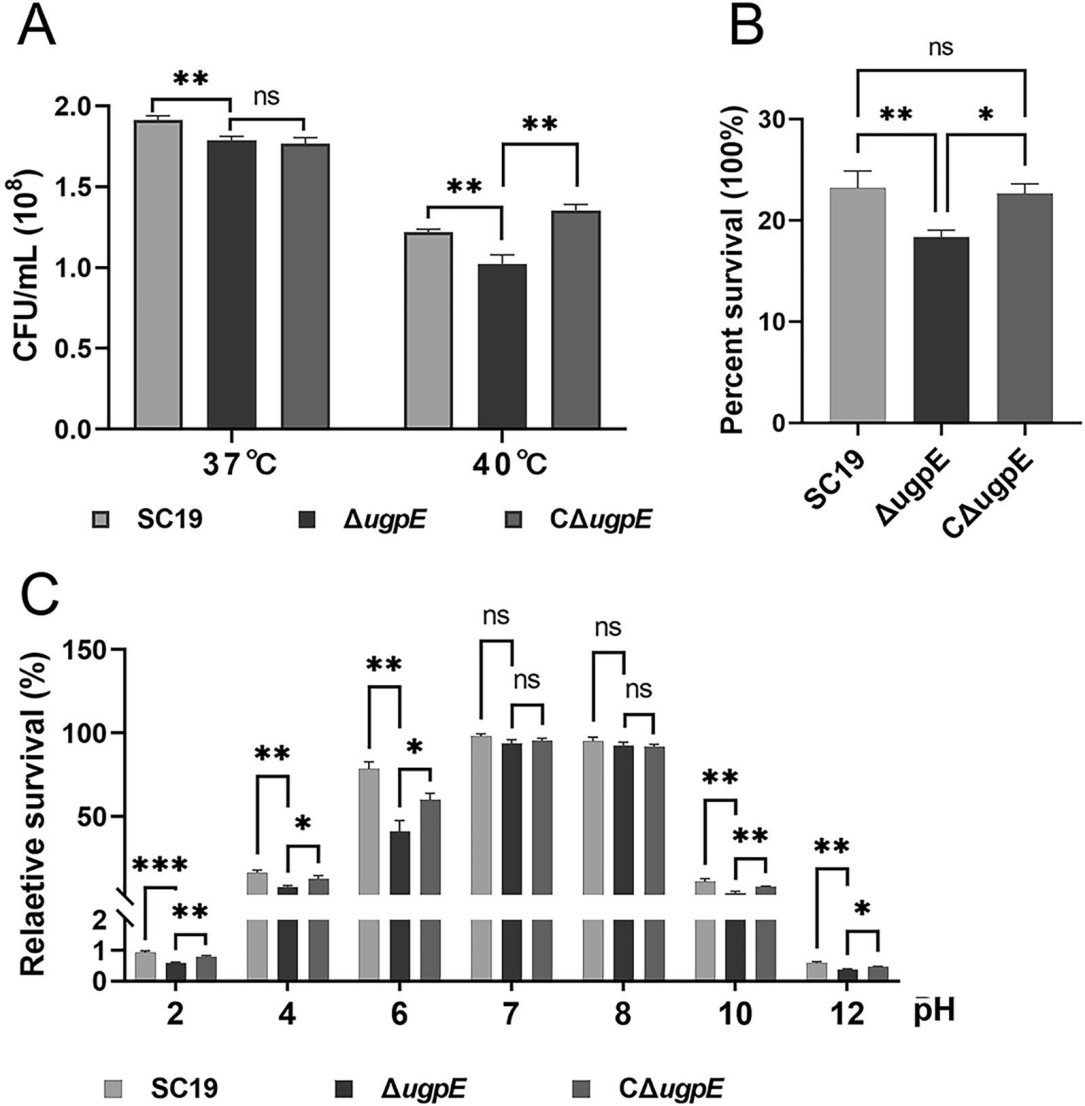
***ugpE*** **deletion led to decreased tolerance to stress.** **A** Heat stress assay of SC19 wild-type, Δ*ugpE* and CΔ*ugpE* at 37 °C and 40 °C. **B** Oxidative stress assay of each group of cells. Cells were treated with hydrogen peroxide (30 mM). **C** Acid–base stress assay of each group of cells. The data are presented as mean ± SD. Data were analyzed using one-way ANOVA (Dunnett test). ***P < 0.001; **P < 0.01; *P < 0.05. ns, not significant.

In a series of processes, such as survival and pathogenesis, pathogens must face various stresses from constantly changing environments, such as acid–base tolerance and hydrogen peroxide. The anti-stress ability directly determines bacterial survival. As shown in Figure 2A, the number of recovered viable bacteria in the Δ*ugpE* mutant was significantly lower than that in the SC19 wild-type at both 37 °C and 40 °C (Figure 2A). Similarly, the Δ*ugpE* mutant was more sensitive to hydrogen peroxide (30 mM) than the SC19 wild-type and complemented strain CΔ*ugpE* (Figure 2B), indicating that the antioxidant capacity of SS2 was downregulated after *ugpE* deletion. Moreover, the acid–base tolerance test shows that the survival ability of the Δ*ugpE* mutant was significantly lower than that of the SC19 wild-type and complemented strain CΔ*ugpE* at all indicated pH points, except at pH 7.0 and pH 8.0, where no differences were observed among the groups (Figure 2C). Overall, these results suggesting that *ugpE* may contribute to bacterial resistance to temperature adaptability at 40 °C, hydrogen peroxide, and acid–base tolerance.

### *ugpE* deletion upregulated SS2 biofilm formation

**Figure 3:**
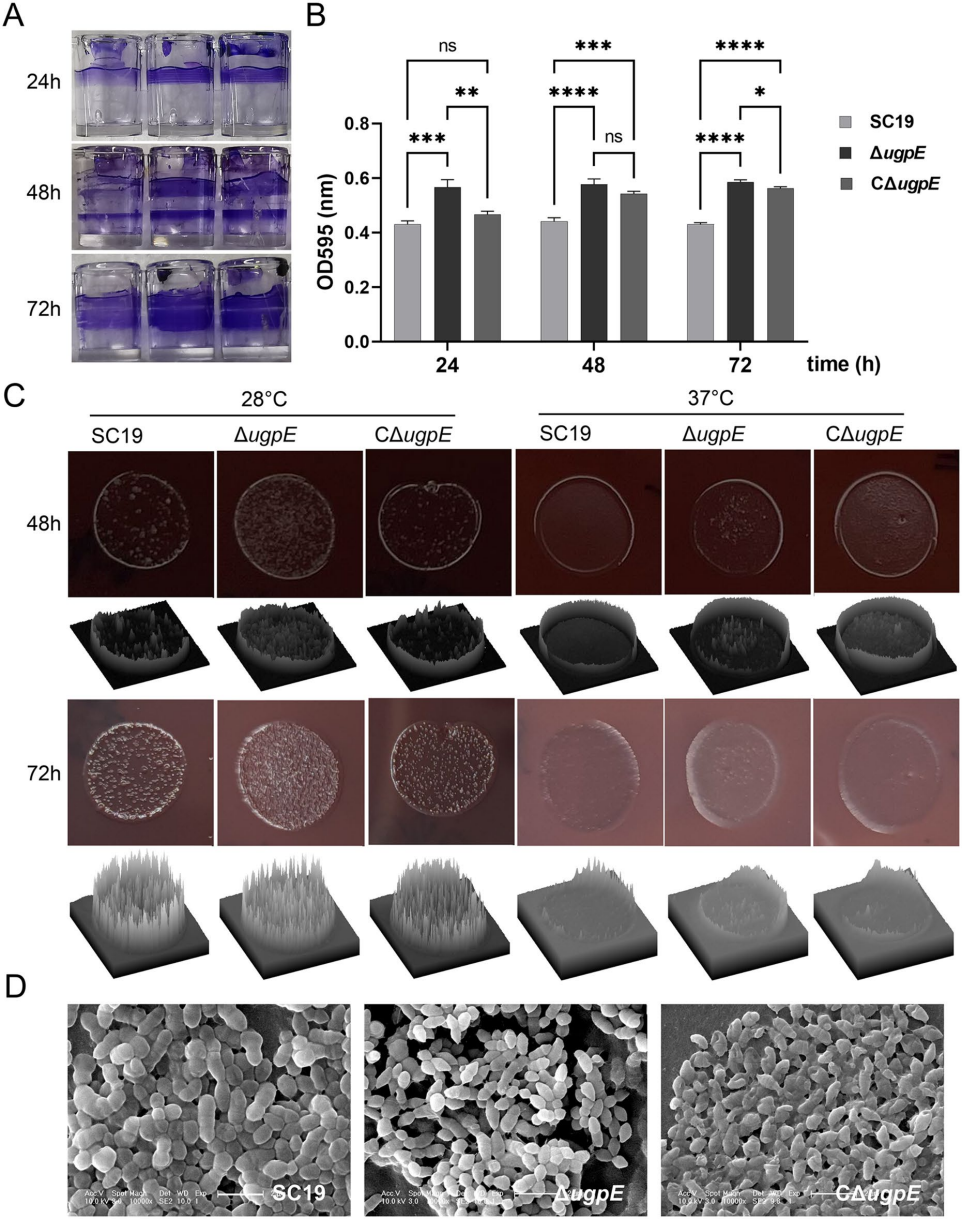
***ugpE*** **deletion up-regulated SS2 biofilm formation.** **A** Observation of biofilms at the air–liquid interface formation on the 96-well plate polystyrene surface after 24 h, 48 h and 72 h of incubation at 37 °C. Adherent cells were stained with 0.2% crystal violet. **B** Quantification of biofilms by dissolution in 30% acetic acid and measurement of absorbance at OD_595_. **C** Analysis of rdar macrocolony morphotype by Congo red agar plate assay after 48 h and 72 h of incubation at 28 °C and 37 °C. The surface morphology of the colonies was plotted and analyzed by ImageJ software (1.8.0). **D** Cell morphology analysis by scanning electron microscopy (SEM) of from plate-grown colonies. Size bar, 2 μm. The data are presented as mean ± SD. Data were analyzed using one-way ANOVA (Dunnett test). ***P < 0.001; **P < 0.01; *P < 0.05. ns, not significant.

Biofilm formation represents a protective mode of growth that enables pathogens to survive in hostile environments [60]. Since deletion of *ugpE* led to decreased anti-stress ability, we wondered if biofilm formation by SC19 was also affected by *ugpE.* We observed that the biofilms were mainly formed as a ring at the air–liquid interface (Figure 3A). Interestingly, the biofilms of the Δ*ugpE* mutant displayed a significantly higher level of adherence to the abiotic polystyrene wall of the well surface after 24 h, 48 h, and 72 h of incubation in standing culture at 37 °C compared to the SC19 wild-type and complemented strain CΔ*ugpE* (Figures 3A and B). We also detected the effect of *ugpE* on rdar (red, dry, and rough) macrocolony morphotypes using a Congo red agar plate assay. Congo red binds to polysaccharides containing contiguous β-(1 → 4)-linked _D_-glucopyranosyl units and β-(1 → 3)-_D_-glucans and has been used to identify exopolysaccharides in biofilms [61]. We found that the SC19 wild-type shows a dry and rough morphotype on Congo red agar plates after 48 h and 72 h at 28 °C, whereas it was a light red and smooth colony at 37 °C (Figure 3C), suggesting that SC19 produces more extracellular matrix at 28 °C than at 37 °C. Notably, a drier and rougher morphotype was observed in the Δ*ugpE* mutant compared to the SC19 wild-type and complemented strain CΔ*ugpE* at both temperatures (Figure 3C), suggesting *ugpE* may be involved in the production of the extracellular matrix of SS2. To our knowledge, this is the first description of regulation of the SS2 rdar morphotype at the gene level. In agreement with the colony morphotype, SEM observation of the agar-grown colonies demonstrates that the production of extracellular matrix was upregulated in the Δ*ugpE* mutant compared to the SC19 wild-type and complemented strain CΔ*ugpE* (Figure 3D).

### *ugpE* deletion impaired SS2 virulence in vitro

**Figure 4:**
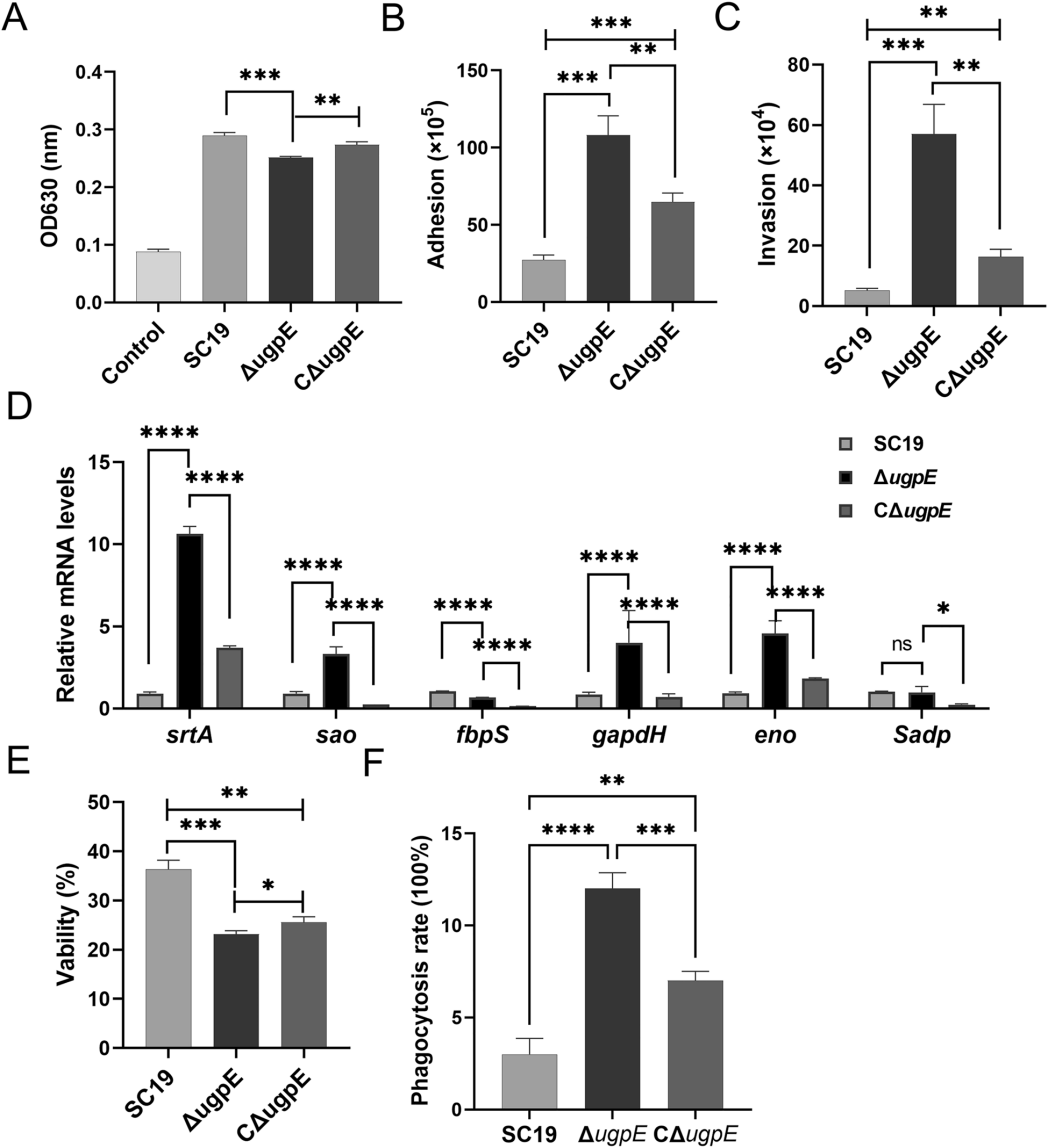
**In vitro virulence after** ***ugpE*** **deletion.** **A** Analysis of the hemolysin activity of SC19 wild-type, Δ*ugpE* and CΔ*ugpE* in 5% sheep blood. **B**, **C** Analysis of adhesion (**B**) and invasion (**C**) abilities of each group of cells to hCMEC/D3. **D** Analysis of mRNA levels of adhesion related genes *srtA*, *sao*, *fbpS*, *gapdh*, *eno*, and *Sadp* by qRT-PCR. **E** Survival in whole human blood of each group of cells. Cells were incubated with germ-free human whole blood at 37 °C for 2 h. **F** Assessment of anti-phagocytosis ability of each group of cells in porcine alveolar macrophages (PAM). The data are presented as mean ± SD. Data were analyzed using one-way ANOVA (Dunnett test). ***P < 0.001; **P < 0.01; *P < 0.05. ns, not significant.

As *ugpE* seems to affect the capsule production of SS2 (Figures 1F–H), we assumed that the virulence phenotypes of SS2 might also be affected. We first checked hemolysin activity and found that the hemolysin ability of the Δ*ugpE* mutant was significantly decreased compared to that of the SC19 wild-type and complemented strain CΔ*ugpE* (Figure 4A).

The ability to adhere to and invade host endothelial cells of the blood–brain barrier (BBB) could play an important role in the development of meningitis caused by *S. suis* [52, 62]. Subsequently, we investigated the adhesion and invasion of each group of strains into human microvascular endothelial cells (hCMEC/D3). Compared to the SC19 wild-type and complemented strain CΔ*ugpE*, we found that the number of both adhered and invaded Δ*ugpE* mutant cells to hCMEC/D3 was significantly higher (Figures 4B and C). With this finding, the transcription levels of adhesion-related genes, including *srtA*, *sao*, *gapdh,* and *eno* also increased compared to the SC19 wild-type and complemented strain CΔ*ugpE*, while the levels of *fbps* and *Sadp* decreased and remained unchanged, respectively (Figure 4D). These results indicate that *ugpE* may be involved in the regulation of bacterial adhesion and invasion of host cells.

Bacterial survival in host blood and anti-phagocytosis ability are critical for immune evasion and the establishment of infection. We further assessed these abilities using whole-blood survival and PAM-mediated phagocytosis analyses. Compared to the SC19 wild-type, of which approximately 36% have survived after whole blood killing, the viability of the Δ*ugpE* mutant and the complemented strain CΔ*ugpE* declined to 21% and 25%, respectively (Figure 4E). In line with this result, we found that the phagocytosis rate of the Δ*ugpE* mutant by PAM cells was significantly higher than that of the SC19 wild-type and complemented strain CΔ*ugpE* (Figure 4F), suggesting that the decreased survival ability of the Δ*ugpE* mutant in human blood was possibly due to an increased susceptibility to PAM phagocytosis.

### *ugpE* deletion attenuated SS2 full virulence in vivo

**Figure 5:**
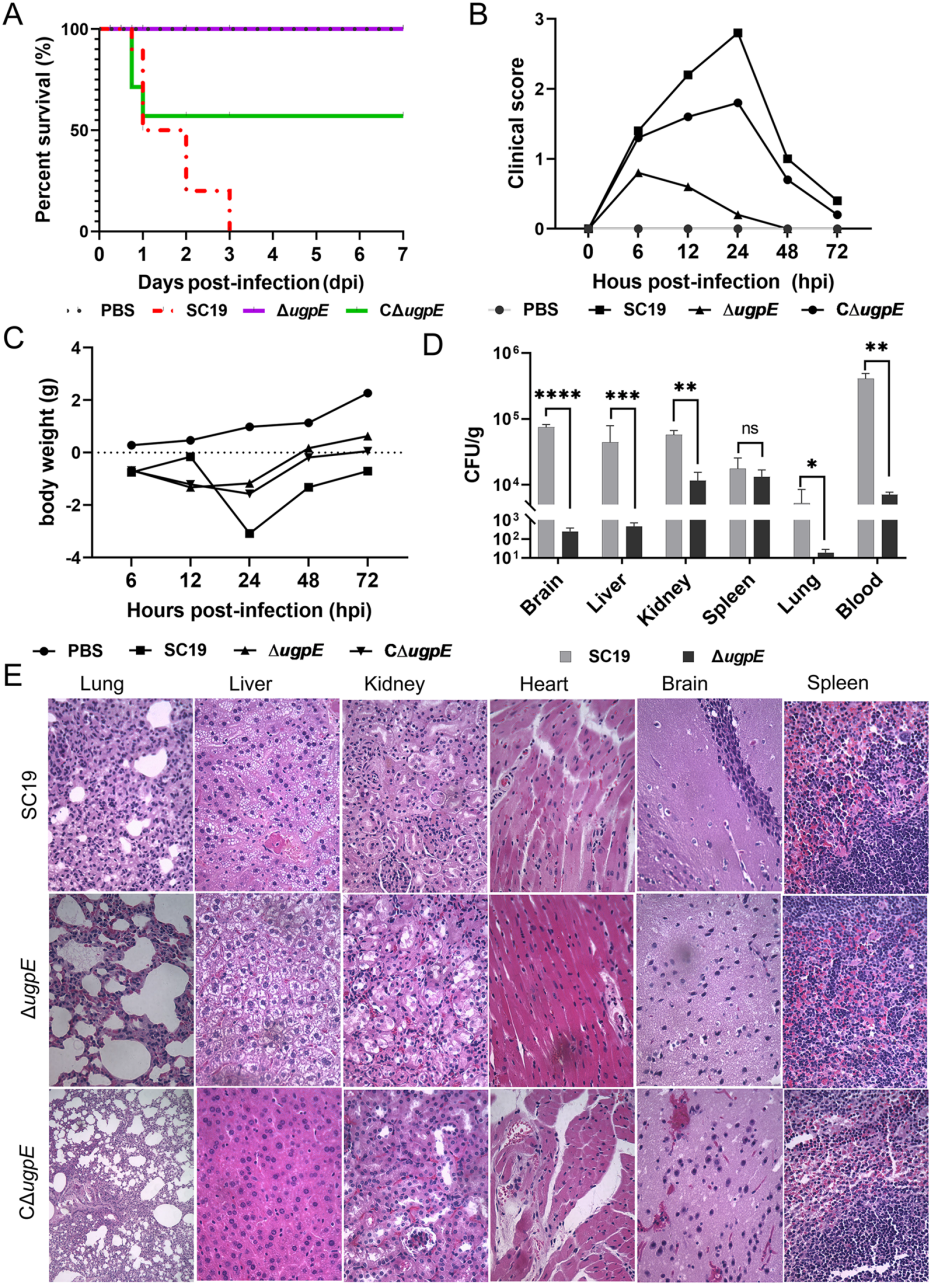
**Determination of bacterial virulence after** ***ugpE*** **deletion in mice.** **A**–**C** Survival curves (**A**), clinical scores (**B**), and body weight changes (**C**) of the mice infected with SC19 wild-type, Δ*ugpE* and CΔ*ugpE*. (D) Bacterial counts in the major organs and blood of mice after infection with each group of cells. The data are presented as mean ± SD. Data were analyzed using paired *t* tests. ***P < 0.001; **P < 0.01; *P < 0.05. ns, not significant. **E** Pathological changes in major organs upon infection with SC19 wild-type, Δ*ugpE* and CΔ*ugpE* strains. The tissue slices were stained with hematoxylin and eosin (HE) and the histopathological changes are indicated with black arrows. Scale bar, 400 ×.

To determine the effect of *ugpE* on the virulence of SS2 in vivo, an SS2 infection model was constructed using BALB/c mice, and the survival of the mice was assessed. The results showed that all the mice died within 3 days of infection by the SC19 wild-type, while no mice or only half of the mice died from infection by the Δ*ugpE* mutant and complemented strain CΔ*ugpE*, respectively (Figure 5A), suggesting *ugpE* is possibly required for SS2 virulence. Furthermore, compared to the SC19 wild-type and CΔ*ugpE* infection groups, mice infected with the Δ*ugpE* mutant had significantly lower clinical scores after 12 h of infection (Figure 5B), indicating dramatically relieved virulence of SS2 upon *ugpE* deletion. In terms of body weight, the three groups of infected mice show similar trends, but the weight loss in the Δ*ugpE* mutant infection group was significantly lower than that in the SC19 wild-type and complemented strain CΔ*ugpE* (Figure 5C). Moreover, the bacterial load in the blood and organs including the brain, liver, kidney, and lung, except in the spleen, was significantly reduced after 24 h of infection with the Δ*ugpE* mutant compared to the SC19 wild-type (Figure 5D), indicating that the colonization ability of SC19 in mice was reduced after *ugpE* deletion, and it was easier to be cleared by the host immune system.

In line with these observations, the histopathological analysis of each organ revealed that the Δ*ugpE* mutant infected mice show weaker pathological changes compared to mice infected with wild-type SC19 and CΔ*ugpE* (Figure 5E; Additional file 5). Specifically, infection with SC19 wild-type and CΔ*ugpE* led to infiltration of inflammatory cells, including monocytes and neutrophils, in all tissues, especially in the lungs, kidneys, brain, and spleen. Moreover, edema and hyperemia were observed in these tissues upon SC19 wild-type and CΔ*ugpE* infection. In contrast, infection with the Δ*ugpE* mutant alleviated the degree of infiltration of inflammatory cells, edema, and hyperemia, especially in the lungs and brain (Figure 5E; Additional file 5), indicating that deletion of *ugpE* impaired the virulence of SC19. Taken together, these results demonstrate that *ugpE* influence the full virulence of SS2 in mice.

### *ugpE* deletion altered distribution of macrophages in major organs of mice upon infection

**Figure 6:**
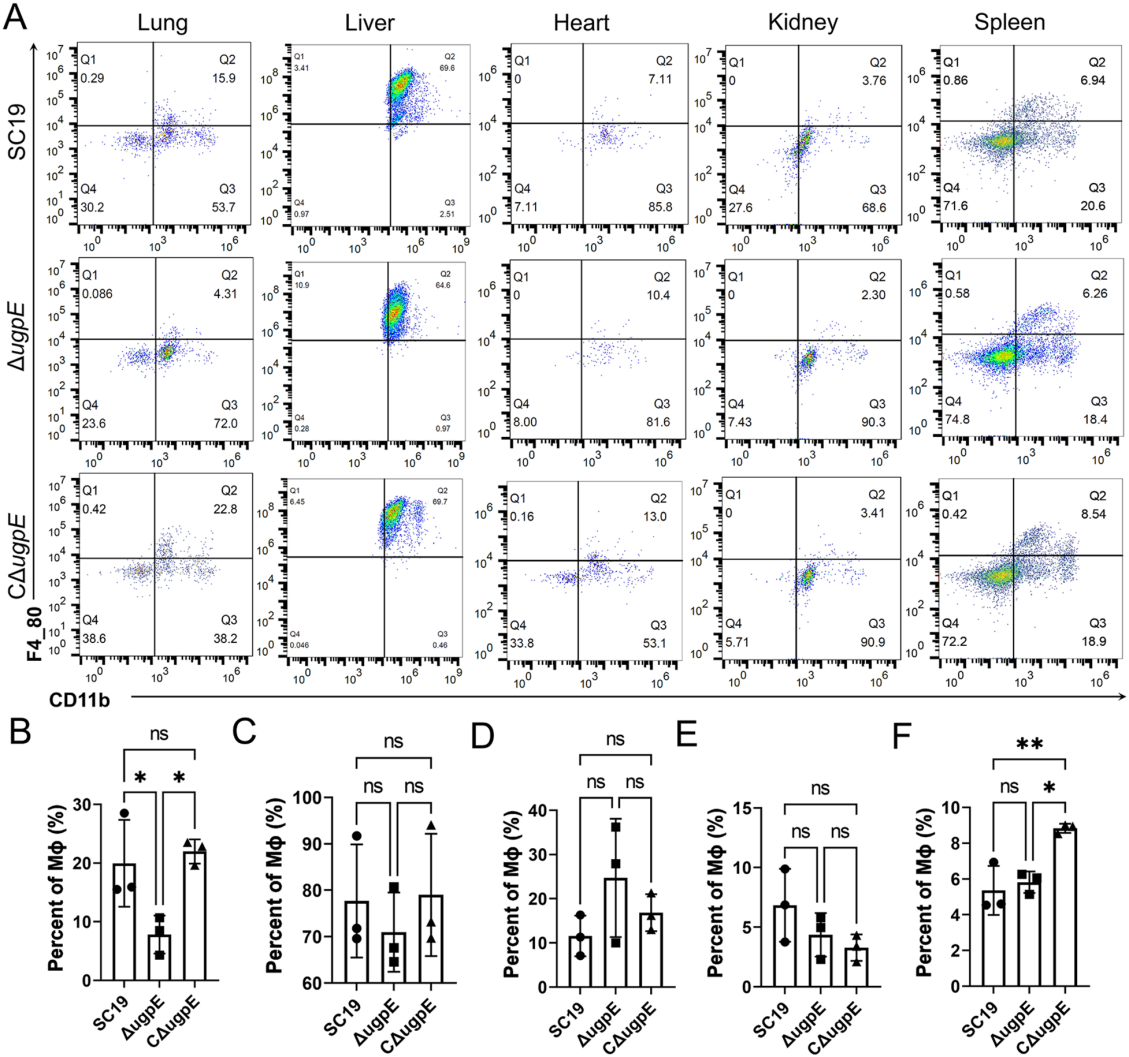
**Distribution of macrophages in major organs of mice upon SC19 infection by flow cytometry**. The data are presented as mean ± SD. Data were analyzed using one-way ANOVA (Dunnett test). ***P < 0.001; **P < 0.01; *P < 0.05. ns, not significant.

Since *ugpE* influenced the full virulence both in vitro and in vivo (Figures 4, 5), we wondered whether the host immune response was also altered upon SS2 infection. The results show that the percentage of macrophages in the lung after 24 h infection with the Δ*ugpE* mutant was significantly lower than that after infection with the SC19 wild-type and CΔ*ugpE*, as demonstrated by flow cytometry (Figures 6A and B). However, the percentage of macrophages in other organs after 24 h infection with the Δ*ugpE* mutant was not significantly different from the other two groups (Figures 6A, C–F).

## Discussion

UgpABCE is a member of the ABC transporter superfamily that is involved in glycerophospholipid synthesis in *E. coli* [23]. In this study, we report a novel role of the Ugp transporter subunit UgpE in the regulation of various cellular phenotypes, including stress tolerance, biofilm formation, and virulence of the zoonotic pathogen *S. suis*. Deletion of *ugpE* impaired cell chain formation, capsular synthesis, and stress tolerance while upregulating biofilm formation and adhesion to hCMEC/D3 cells. Importantly, bacterial virulence was attenuated in mice after deletion of *ugpE*. These results suggest *ugpE* is a novel factor associated with virulence and various cellular phenotypes of *S. suis*.

In SC19, UgpE contains a canonical ABC_TM1 domain (PS50928, ABC transporter integral membrane type-1) profile, six transmembrane domains, as well as conserved residues that possibly bind with ATPase UgpC (Additional file 6). In *E. coli*, UgpE contributes to the transport of both G3P and glycerophosphodiesters from the periplasm to cytosol under phosphate starvation conditions [23, 27]. Glycerophosphodiesters are degraded into G3P and further acylated by *sn*-glycerol-3-phosphate acyltransferase to initiate the biosynthesis of membrane glycerophospholipids or central carbon metabolism [63, 64]. Indeed, glycerophosphodiesters are also taken up by Glp (glycerol-3-phosphate transporter), apart from the UgpABCE transporter system [65]. However, preliminary analysis demonstrated that no Glp homolo was present in the SC19 genome (data not shown), indicating that the UgpABCE transporter might be the sole system for G3P and glycerophosphodiester acquisition in SC19. Interestingly, we did not observe any obvious morphological changes in the cell membranes or cell walls among the groups (Figures 1F and H). Considering the importance of glycerol phosphate in membrane phospholipid synthesis, we hypothesized that other cell wall components might be impaired by *ugpE* deletion. To the best of our knowledge, this is the first study to identify a potential role of UgpE on regulation of various cellular phenotypes of Gram positive bacterium *S. suis*.

Although the cell membrane and cell wall were hardly affected, the capsule structure almost disappeared (Figures 1F–G). The capsule is a protective polysaccharide layer around the cell, which is an important virulence factor for most encapsulated streptococci. The absence of CPS correlates with increased phagocytosis by macrophages and decreased virulence in murine and pig models of infection [66]. In SS2, the capsular locus (*cps*) responsible for CPS biosynthesis involves 25 genes [59]. It has been shown that inactivation of the *cps2B* gene for polysaccharide chain length determination, *cps2EF*, *cps2G*, *cps2J* and *cps2L* genes for glycosyltransferases leads to impaired capsule production [7, 67]. *cpsBCD* genes form a tyrosine phosphoregulatory system that controls the CPS assembly machinery, of which CpsB assists in CpsD dephosphorylation and its interaction with CpsC, leading to elevated CPS polymerization [68, 69]. The *cpsPQRST* genes show homology to the NeuBCDA proteins of Group B *Streptococcus* (GBS) and *E. coli* and thus have been predicted to be involved in the synthesis of sialic acid (NeuNAc), one of the main components of the CPS of *S. suis* [59]. Inactivation of *neuC* (*cpsQR*) results in the loss of the whole capsule of *S. suis*, decreasing the anti-phagocytosis ability of macrophages, and impairing virulence in mice [70]. In GBS, *neuD* functions as a Sia O-acetyltransferase and is required for the synthesis of the unique sialylated CPS shared only by *S. suis* and GBS [71, 72]. In this study, a reduction in the transcription levels of *cps-2B*, *cps-2C* and *cps-2S* was observed in the Δ*ugpE* mutant (Figure 1I), suggesting that the unencapsulated phenotype of the Δ*ugpE* mutant may be due to interference with polysaccharide chain formation and the expression of sialic acid for CPS synthesis in *S. suis*. In line with these observations, the unencapsulated Δ*ugpE* mutant exhibited a lower survival rate in whole human blood, antiphagocytic ability against PAM, and decreased virulence in mice compared to the SC19 wild-type (Figures 4E and F, 5), suggesting that deficiency in CPS development is a critical reason for the weakened virulence of *S. suis* by *ugpE* deletion.

The CPS is synthesized by the Wzx/Wzy pathway in *S. suis* [73, 74]. Moreover, the CPS synthesis is also regulated by small RNA (sRNA) in *S. suis* [75, 76]. sRNA rss04 contributes to meningitis induction in mice by regulating CPS synthesis [75]. Deletion of sRNA23 leads to shorter chain length, thinner CPS, and weaker biofilm formation ability in *S. suis* 05ZYH33 [76]. In this study, we identified for the first time that UgpE, which belongs to the Ugp transporter system, is involved in the regulation of CPS synthesis in Gram positive streptococci.

Notably, both the adhesion and invasion abilities of host hCMEC/D3 cells were enhanced in the Δ*ugpE* mutant compared to those in the SC19 wild-type (Figures 4B and C). We speculated that although CPS is a critical antiphagocytic factor that resists phagocytosis by macrophages and neutrophils in the blood, it may interfere with the adhesion and invasion of host cells by hindering cell wall components, especially some vital virulence factors located on the cell surface for adhesion. Indeed, many studies have demonstrated that the repression of CPS production in SS2 enhances the adhesion and invasion of host cells and impairs virulence in mice [75, 77, 78]. As anticipated, the transcription levels of genes encoding cell surface virulence factors, including *srtA*, *sao*, *gapdh*, and *eno* were upregulated in the Δ*ugpE* mutant compared to those in the parental strain (Figure 4D). These results suggest that impairment of CPS by the Δ*ugpE* mutant may lead to exposure of cell surface proteins, which contributes to bacterial adhesion and invasion of host cells. In contrast, impairment in CPS decreased antiphagocytic ability against macrophages and neutrophils in the blood, resulting in attenuated virulence, as confirmed by the mouse infection model (Figure 5).

Biofilm formation represents a protected mode of growth that renders bacterial cells less susceptible to host immune responses and thus enables pathogens to survive in hostile environments [60]. Interestingly, the Δ*ugpE* mutant exhibited enhanced biofilm formation on the polystyrene surface, along with decreased survival in whole human blood and anti-phagocytic ability in PAM (Figures 3A and B, 4E and F). Considering the critical role of CPS in the resistance to phagocytosis, we reasoned that infection by the Δ*ugpE* mutant may not be established due to the loss of CPS structure, despite the upregulation of biofilm formation. Indeed, studies have shown that the CPS structure affects bacterial biofilm formation. In *S. pneumoniae*, encapsulated clinical pneumococcal isolates are impaired in their capacity to form biofilms, and reduction in the amount of CPS is usually correlated with increased biofilm-forming capacity [79]. In SS2, the flavonoid compound rutin specifically downregulated biofilm formation by interfering with CPS biosynthesis [80].

In summary, our study reported for the first time that UgpE, which belongs to the Ugp transporter system, influence the full virulence and biofilm formation of the zoonotic pathogen *S. suis*. UgpE may contribute to bacterial virulence by positively regulating CPS synthesis, demonstrating that UgpE is a novel virulence-associated factor of *S. suis*. Our results expand the current knowledge on the pathogenesis of *S. suis*.

## Supplementary Information

**Additional file 1. Bacterial strains and plasmids used in this study**. Detailed bacterial strains and plasmids information are provided.

**Additional file 2. Primers used in this study**. Primer sequences are listed.

**Additional file 3. Construction of** ***ugpE*** **deletion mutant and complemented strains in SS2.** (A) Construction of pSET4s::Δ*ugpE* plasmid for *ugpE* deletion. M, marker; 1, pSET4s::Δ*ugpE*; 2, positive samples; 3, ddH_2_O. Expected size, 1531 bp. (B) Identification of *ugpE* deletion mutant. M, marker; 1, SC19 wild-type; 2-3, negative samples; 4, ddH_2_O; 5-8, positive samples. Expected size, 643 bp. (C) Construction of pSET2::Δ*ugpE* plasmid for *ugpE* complementation. M, marker; 1-5, samples; 6, SC19 pSET2::Δ*ugpE*; 7, ddH_2_O. Expected size, 888 bp. (D) Identification of *ugpE* complemented strain. M, marker; 1-3, positive samples; 4, SC19 pSET2::Δ*ugpE*. Expected size, 888 bp.

**Additional file 4. Observation of cell morphology by microscopy**. (A) Growth kinetics of SC19 wild-type, Δ*ugpE* and CΔ*ugpE* at 37 °C. OD_600_ values were measured every hour for a total of 15 h. (B) Observation of cell morphology by light microscopy (1000 ×). (C) Analysis of cell number per chain in each group of cells. The quantification is based on results from at least three independent experiments with the assessment of 200 cells from each group. (D) Observation of cell morphology by transmission electron microscopy (TEM). Size bar, 1 μm. (E) Analysis of cell wall thickness in each group of cells. The quantification is based on results from at least three independent experiments with the assessment of 20 cells from each group. (F) Analysis of heat tolerance at 37 °C. The data are presented as mean ± SD. Data were analyzed using two-way (A and C) and one-way ANOVA (Dunnett test) (E and F), respectively. ***P < 0.001; **P < 0.01; *P < 0.05. ns, not significant.

**Additional file 5. Pathological changes in major organs upon infection with SC19 wild-type, Δ*ugpE*** **and CΔ*ugpE*** **strains**. The tissue slices were stained with hematoxylin and eosin (HE). Scale bar, 100×.

**Additional file 6. Bioinformatic analysis of UgpE of** ***S. suis*** **SC19**. (A) The graphical representation and schematic indication of the positions of the conserved residues (indicated by the red dash, blue and orange rods) in the ABC_TM1 domain of the 295 aa UgpE from *S. suis* SC19. The graph was assessed by ExPASy_Prosite. (B) Transmembrane domain prediction by TMHMM posterior. (C) Predicted structural model of the UgpE from *S. suis* SC19 using AlphaFold.

## Data Availability

The datasets supporting the conclusions of this article are available in the Figshare (https://doi.org/10.6084/m9.figshare.27094336.v1).
