# Supplementary material for: Identification and characterization of ugpE associated with the full virulence of Streptococcus suis
Source: Vet Res. 2025 Apr 16;56:82. doi: 10.1186/s13567-025-01513-z (PMC12001685; doi:10.1186/s13567-025-01513-z)
Supplement: Supplementary file 1 — Additional file 1. Bacterial strains and plasmids used in this study. Detailed bacterial strains and plasmids information are provided. [file 13567_2025_1513_MOESM1_ESM.docx]

**Additional file 1 Bacterial strains and plasmids used in this study**

| Strains | Description^a^ | Source |
| --- | --- | --- |
| *S. suis* SC19 | *S. suis* serotype 2, ST7, *mrp^+^ef^+^sly^+^* | This study |
| *E. coli* DH5α | λφ80d*lacZ* ΔM15 Δ(*lacZYA*-argF)U169  *recA1* *endA1 hsdR17*(rK- mK- ) *supE44*  *thi-1 gyrA relA1* | New England Biolabs |
| *S. suis* Δ*ugpE* | The isogenic *ugpE* deletion mutant of SC19 | This study |
| SC19 CΔ*ugpE* | The complementary strain | This study |
| Plasmids |  |  |
| pSET2 | *E. coli* - *S. suis* shuttle vector, Spc^r^ | This study |
| pSET4s | *E. coli* - *Streptococcus* shuttle cloning vector,  Spc^r^ | This study |
| pSET2::Δ*ugpE* | pSET2 inserted with the intact *ugpE* and the upstream promoter, Spc^r^ | This study |
| pSET4s::Δ*ugpE* | pSET4s carrying the construct for *ugpE* allelic replacement, Spc^r^ | This study |

^a^ Spc^r^, spectinomycin resistance
