# Supplementary material for: Identification and characterization of ugpE associated with the full virulence of Streptococcus suis
Source: Vet Res. 2025 Apr 16;56:82. doi: 10.1186/s13567-025-01513-z (PMC12001685; doi:10.1186/s13567-025-01513-z)
Supplement: Supplementary file 2 — Additional file 2. Primers used in this study. Primer sequences are listed. [file 13567_2025_1513_MOESM2_ESM.docx]

**Additional file 2 Primers used in this study**

| Primers | Sequence (5’-3’) |
| --- | --- |
| Gene deletion | |
| A1 | CCAAGCTTAATAAAACCATCAATAAACAGTT |
| A2 | TTTCTATAAAAATTTTGAACAATTACGAACCTCCTATAAGCCTTGTCC |
| A3 | AAAGATATTTTTAAAACTTGTTAATGCTTGGAGGATATTCGGAACAGG |
| A4 | CGGAATTCAATGTTAGCTTTATCCCAAGTGAAGT |
| Gene cloning | |
| Y1 | CATGCATGCCATGAATACACAAATGTACAC |
| Y2 | CGCGGATCTTAACCTTTTAGTGAACCAG |
| Confirmatory primers | |
| A5 | ACACACCTTATAGTAGAACCGTGCC |
| A6 | CCGTAGTTTCATTCTTAAGGTA |
| qRT-PCR primers | |
| srta-F | GTGGGATGCACAACGCTTAC |
| srta-R | GGCTGGCCAACGCATAATTT |
| sao-F | TTTGGTCGTGAGCTTCTAG |
| sao-R | CCAGGCTTATCGAATGATAG |
| fbps-F | GGTGGCCCAGCAGGCCAATG |
| fbps-R | CCGCCAATCCCTGCTCCTGC |
| gadph-F | AGAAGTAAACGCTGCTAT |
| gadph-R | CAAACAATGAACCGAAT |
| eno-F | CGTGATCAACAAGCTATC |
| eno-R | GTGTAAAGTGGCACTTCAA |
| sade-F | GTAGAGAAGCAACAGTCGCC |
| sade-R | GTTACTTTCCACTGAATCTG |
| Cps2b-F | ATGAACAATCAAGAAGTAAATGCAA |
| Cps2b-R | CTATTTTAATTTCTTCGAATCTGGT |
| Cps2c-F | ATGGCGATGTTAGAAATTGCACGTA |
| Cps2c-R | TTAGGCTTTTTTGCCGTAATTTCCG |
| Cps2r-F | ATGAAAAAAGTAGCCTTTCTAGGAG |
| Cps2r-R | CTATTTAATCTTTCTAGCAGGTACA |
| Cps2m-F | GTGCGTTCCAAGGTAGATACTTTCA |
| Cps2m-R | TTATCGTTTTCCACGTACTCTCATA |
| Cps2s-F | ATGGAACCAATTTGTCTGATTCCTG |
| Cps2s-R | TTATCTTGTCAAACTTGTCAAAATC |
