# Supplementary figures and images for: Identification and characterization of ugpE associated with the full virulence of Streptococcus suis
Source: Vet Res. 2025 Apr 16;56:82. doi: 10.1186/s13567-025-01513-z (PMC12001685; doi:10.1186/s13567-025-01513-z)

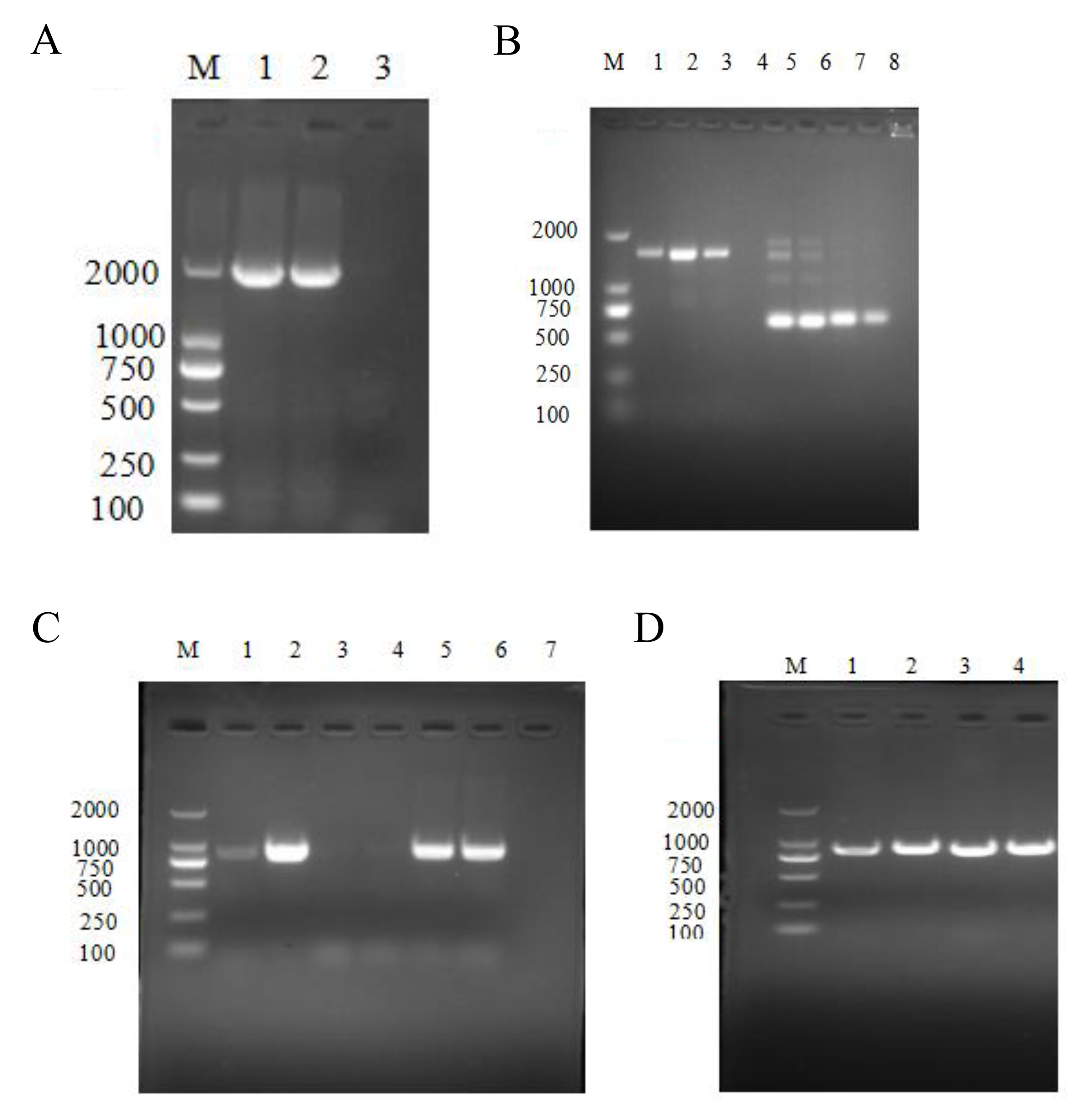

Supplement: Supplementary file 3 — Additional file 3. Construction of ugpE deletion mutant and complemented strains in SS2. (A) Construction of pSET4s::ΔugpE plasmid for ugpE deletion. M, marker; 1, pSET4s::ΔugpE; 2, positive samples; 3, ddH2O. Expected size, 1531 bp. (B) Identification of ugpE deletion mutant. M, marker; 1, SC19 wild-type; 2-3, negative samples; 4, ddH2O; 5-8, positive samples. Expected size, 643 bp. (C) Construction of pSET2::ΔugpE plasmid for ugpE complementation. M, marker; 1-5, samples; 6, SC19 pSET2::ΔugpE; 7, ddH2O. Expected size, 888 bp. (D) Identification of ugpE complemented strain. M, marker; 1-3, positive samples; 4, SC19 pSET2::ΔugpE. Expected size, 888 bp. [file 13567_2025_1513_MOESM3_ESM.tif]

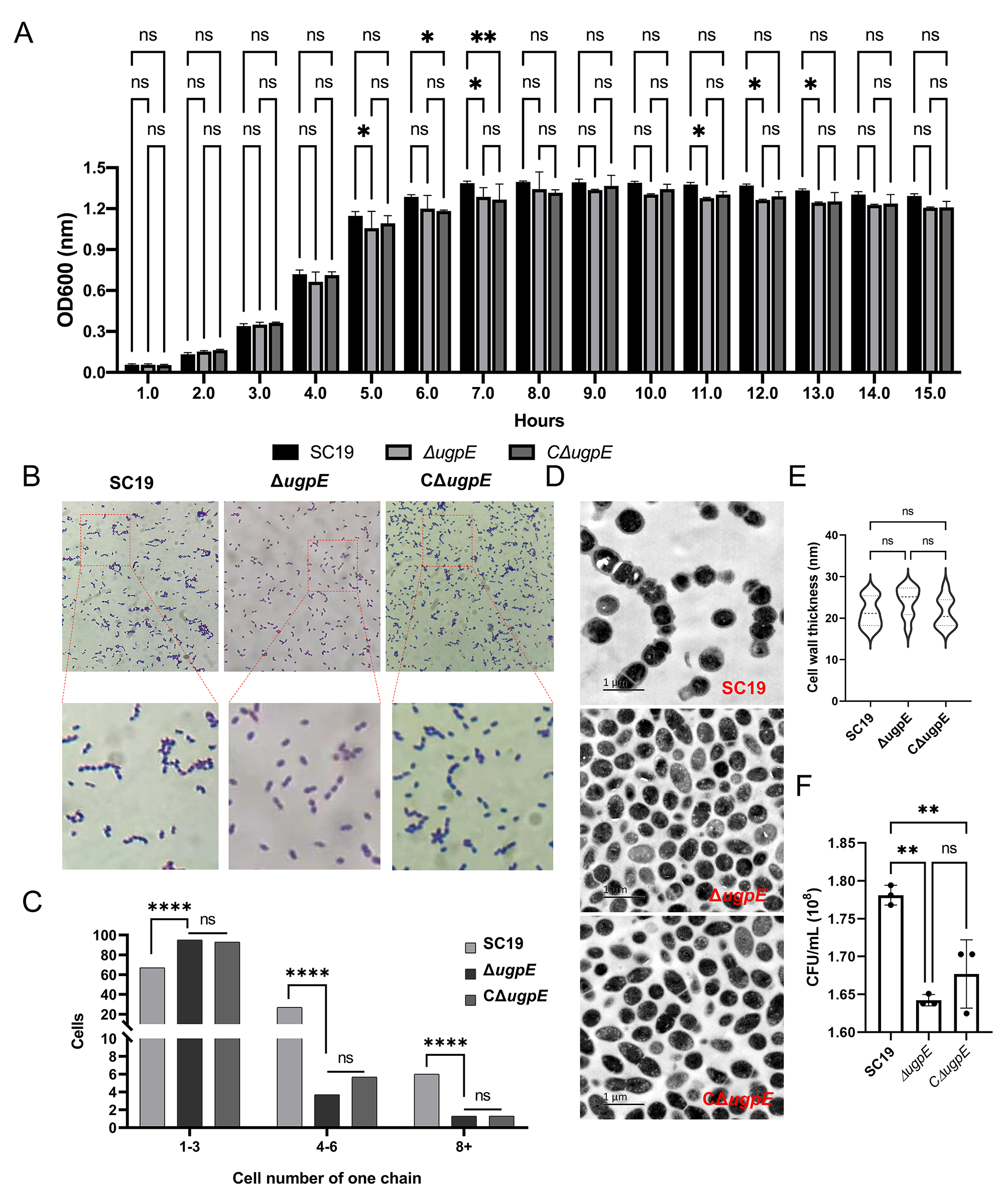

Supplement: Supplementary file 4 — Additional file 4. Observation of cell morphology by microscopy. (A) Growth kinetics of SC19 wild-type, ΔugpE and CΔugpE at 37 °C. OD600 values were measured every hour for a total of 15 h. (B) Observation of cell morphology by light microscopy (1000 ×). (C) Analysis of cell number per chain in each group of cells. The quantification is based on results from at least three independent experiments with the assessment of 200 cells from each group. (D) Observation of cell morphology by transmission electron microscopy (TEM). Size bar, 1 μm. (E) Analysis of cell wall thickness in each group of cells. The quantification is based on results from at least three independent experiments with the assessment of 20 cells from each group. (F) Analysis of heat tolerance at 37 °C. The data are presented as mean ± SD. Data were analyzed using two-way (A and C) and one-way ANOVA (Dunnett test) (E and F), respectively. ***P < 0.001; **P < 0.01; *P < 0.05. ns, not significant. [file 13567_2025_1513_MOESM4_ESM.tif]

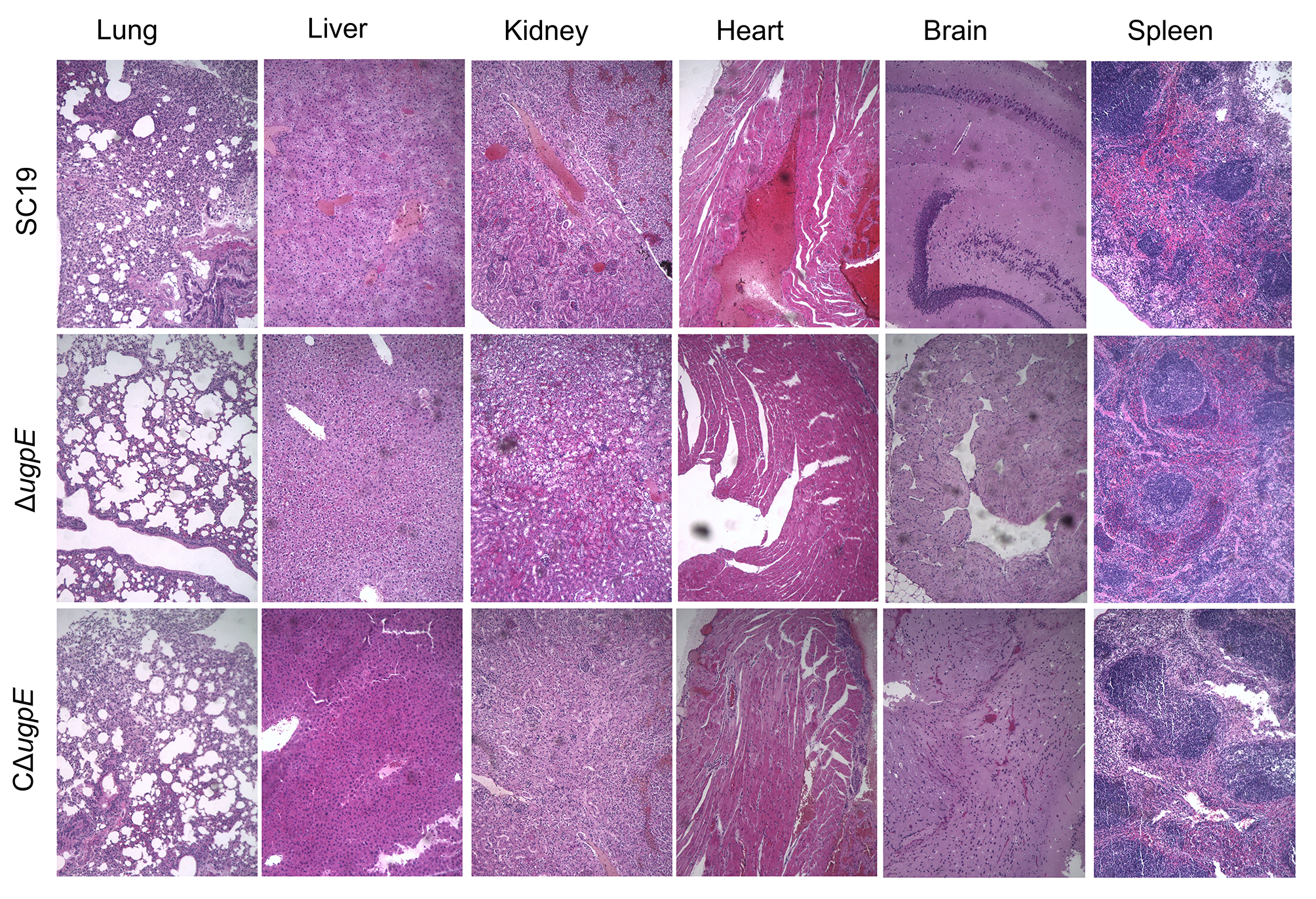

Supplement: Supplementary file 5 — Additional file 5. Pathological changes in major organs upon infection with SC19 wild-type, ΔugpE and CΔugpE strains. The tissue slices were stained with hematoxylin and eosin (HE). Scale bar, 100×. [file 13567_2025_1513_MOESM5_ESM.tif]

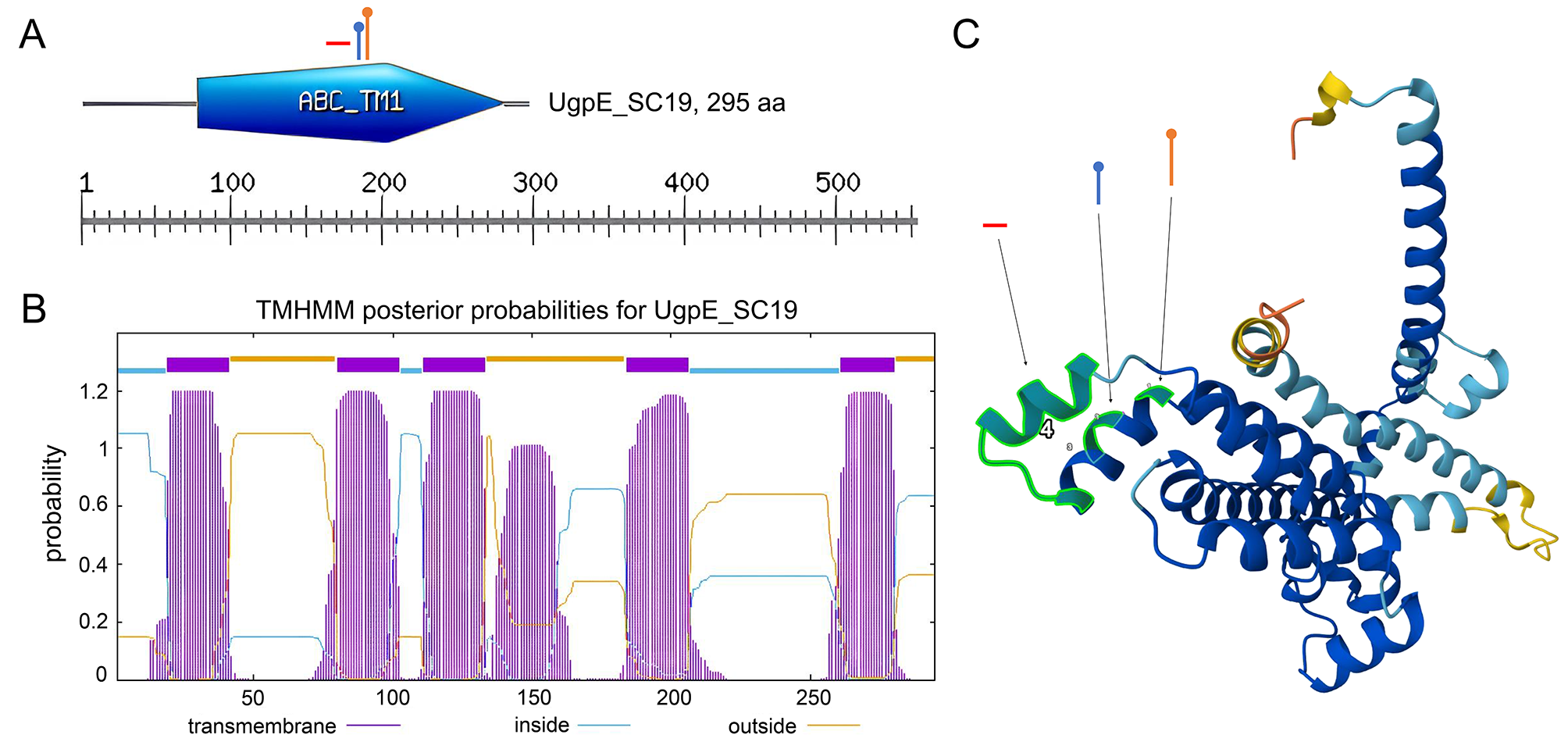

Supplement: Supplementary file 6 — Additional file 6. Bioinformatic analysis of UgpE of S. suis SC19. (A) The graphical representation and schematic indication of the positions of the conserved residues (indicated by the red dash, blue and orange rods) in the ABC_TM1 domain of the 295 aa UgpE from S. suis SC19. The graph was assessed by ExPASy_Prosite. (B) Transmembrane domain prediction by TMHMM posterior. (C) Predicted structural model of the UgpE from S. suis SC19 using AlphaFold. [file 13567_2025_1513_MOESM6_ESM.tif]
